# UBE2O targets Mxi1 for ubiquitination and degradation to promote lung cancer progression and radioresistance

**DOI:** 10.1038/s41418-020-00616-8

**Published:** 2020-09-08

**Authors:** Yumei Huang, Xijie Yang, Yanwei Lu, Ye Zhao, Rui Meng, Sheng Zhang, Xiaorong Dong, Shuangbing Xu, Gang Wu

**Affiliations:** Cancer Center, Union Hospital, Tongji Medical College, Huazhong University of Science and Technology, Wuhan, 430022 China

**Keywords:** Tumour biomarkers, Preclinical research

## Abstract

UBE2O, an E2/E3 hybrid ubiquitin-protein ligase, has been implicated in the regulation of adipogenesis, erythroid differentiation, and tumor proliferation. However, its role in cancer radioresistance remains completely unknown. Here, we uncover that UBE2O interacts and targets Mxi1 for ubiquitination and degradation at the K46 residue. Furthermore, we show that genetical or pharmacological blockade of UBE2O impairs tumor progression and radioresistance in lung cancer in vitro and in vivo, and these effects can be restored by Mxi1 inhibition. Moreover, we demonstrate that UBE2O is overexpressed and negatively correlated with Mxi1 protein levels in lung cancer tissues. Collectively, our work reveals that UBE2O facilitates tumorigenesis and radioresistance by promoting Mxi1 ubiquitination and degradation, suggesting that UBE2O is an attractive radiosensitization target for the treatment of lung cancer.

## Introduction

Lung cancer is the deadliest malignancy worldwide, of which non-small cell lung cancer (NSCLC) represents ~85% [1, 2]. Lung adenocarcinoma is the most common subtype and accounts for 50% of NSCLC [2]. Until now, radiotherapy plays essential roles in the treatment of lung cancer at different stages and can significantly prolong the survival time of patients [3]. However, cancer cell radioresistance is regarded as the leading cause of local recurrence and distant metastasis. Therefore, it is of great theoretical and clinical value to identify novel lung cancer radiosensitization targets.

The ubiquitin-proteasome system (UPS) mediates more than 80% of protein degradation in eukaryotes, which involves ubiquitin, ubiquitin-activating enzyme (E1), ubiquitin-conjugating enzyme (E2s), ubiquitin ligase (E3s), and proteasome [4]. Accumulating evidence has shown that UPS plays a pivotal role in cell proliferation and apoptosis, cell cycle control as well as DNA damage repair [5, 6]. Ubiquitin-conjugating enzyme E2O (UBE2O), an E2/E3 hybrid ubiquitin-protein ligase, is located in the 17q25 region of human chromosomes and displays both E2 ubiquitin conjugating enzyme and E3 ubiquitin ligase activities [7, 8]. UBE2O has been reported to target several proteins for ubiquitination and implicate in chromatin-associated protein nuclear transport, adipogenesis, tumor progression, and metastasis [9–13]. For example, UBE2O binds to and multi-monoubiquitinates BAP1 to induce its cytoplasmic localization and promotes adipocyte differentiation [9]. UBE2O also ubiquitinates SMAD6 and controls the BMP7-induced adipogenesis [10]. It has been documented that UBE2O targets AMPKα2 for degradation to facilitate tumor progression and metastasis [13]. Recently, it was revealed that UBE2O is an independent quality control factor to efficiently targets unassembled orphan proteins and ribosomal proteins for degradation [14, 15]. Nevertheless, it is still unclear whether UBE2O plays an essential role in cancer radioresistance.

MAX interactor 1 (Mxi1) is located in the cancer hotspot region 10q24-q25 of human chromosomes [16]. Mxi1 can antagonize the transcriptional activity of Myc by competing with Max [17], and plays essential roles in multiple biological processes which include cell growth and differentiation, cell cycle regulation, cell apoptosis, and radiosensitivity [18]. Previous study has shown that Mxi1^−/−^ knockout mice are prone to form squamous cell carcinoma and malignant lymphoma [19], indicating that Mxi1 downregulation may contribute to tumorigenesis. Our previous study has found that β-Trcp targets Mxi1 for ubiquitination and destruction in a S6K1-dependent manner [20]. This is the first report showing that Mxi1 is an unstable protein which can be modulated at the posttranscriptional level. Besides, we also found that Mxi1 is downregulated and leads to enhanced radiosensitivity in lung cancer. However, the molecular mechanisms how Mxi1 is regulated has not been fully elucidated.

In this study, we first reveal that UBE2O is physically associated with Mxi1 in vitro and in vivo. We then show that UBE2O targets Mxi1 for ubiquitination and degradation at the K46 residue. Functionally, genetical or pharmacological inhibition of UBE2O significantly impairs lung cancer progression and radioresistance in vitro and in vivo. Clinically, UBE2O is remarkedly upregulated and negatively correlated with Mxi1 downregulation in lung cancer tissues.

## Materials and methods

### Cell culture and plasmids

The human bronchial epithelioid cell line HBE, HEK293T, SMCC-7721, HeLa, and all human lung cancer cell lines including A549, H1299, H292, HCC827, and H1975 cells were purchased from American Type Culture Collection and cultured in RPMI1640 or DMEM with 10% FBS in incubator at 37 °C with 5% CO_2_. All plasmids were subcloned into entry vector and then transferred to destination vector with indicated Myc, HA, SFB, or GST tag for the expression using Gateway Technology (Invitrogen). K46R mutation was generated by the KOD Hot Start DNA Polymerase (Novagen) and validated by DNA sequencing.

### Antibodies and reagents

Anti-UBE2O antibody (GTX119315) for immunohistochemical (IHC) analysis was purchased from GeneTex. Anti-UBE2O antibody (A301-873A) for immunoblotting was purchased from Bethyl Laboratories. Anti-Mxi1 antibody (HPA035319), anti-Flag antibody (F1804), and Cycloheximide (01810) were purchased from Sigma-Aldrich. Anti-Myc (sc-40) and anti-Mxi1 (sc-1042) antibodies were obtained from Santa Cruz Biotechnology. Anti-GAPDH (60004-1-Ig) antibody was obtained from Proteintech. Anti-HA (#3724) and anti-GST (#2624) antibodies were obtained from Cell Signaling Technology. Anti-ATM (A19650) and anti-phospho-ATM-Ser1981 (AP0008) antibodies were obtained from ABclonal. The proteasome inhibitor MG132 (474790) was purchased from Millipore. Arsenic trioxide (ATO) was a clinically available drug which was obtained from Beijing ShuangLu Pharmaceutical Co., Ltd., China (approval number: H20080664).

### RNA interference

Lipofectamine RNAiMAX reagent (Invitrogen) was used for siRNA transfection. After 48 h of transfection, cells were collected and analyzed by western blotting. The siRNAs ON-TARGETplus SMARTpool for Mxi1 were purchased from Dharmacon. The siRNAs targeting UBE2O and β-Trcp were as follows:

si-UBE2O#1: 5-GGUUGUAGAGUUGAAAGUUTT-3;

si-UBE2O#2: 5-CCACCCAGUGUGAAACCAATT-3;

si-β-Trcp: 5-AAGUGGAAUUUGUGGAACAUC-3 [20, 21].

### GST pull-down assay

This assay was carried out as described previously [22, 23]. In brief, SFB-UBE2O plasmid was transfected into HEK293T cells for 24 h, and then the cell lysates were incubated with purified GST-only or GST-Mxi1 fusion proteins plus GST beads (GE Healthcare) overnight. The samples were centrifuged and washed five times with NETN buffer and subjected to SDS-PAGE.

### Western blotting and co-immunoprecipitation assay

Cells were lysed in NETN buffer (100 mM of NaCl, 1 mM of EDTA, 20 mM of Tris HCl, pH 8.0, and 0.5% Nonidet P-40), and then cell lysates were separated by SDS-PAGE. For the exogenous co-immunoprecipitation, cell extracts were incubated with Myc beads (Santa Cruz Biotechnology), S beads (Novagen), or HA-beads (Millipore) overnight. For the endogenous binding, cell lysates were incubated with protein A/G agarose (Santa Cruz Biotechnology) plus IgG or anti-UBE2O/Mxi1 antibody overnight. After being centrifuged and washed with NETN buffer five times, the precipitates were analyzed by immunoblotting.

### In vivo ubiquitination assay

The indicated plasmids and siRNAs were transfected into cells and proteasome inhibitor MG132 was added for 4 h before harvested. Cell lysates were incubated with S protein beads for co-immunoprecipitation, and ubiquitinated Mxi1 was detected by immunoblotting with anti-HA antibody.

### Identification of Mxi1 ubiquitination modification site

SFB-Mxi1, Myc-UBE2O, and HA-ubiquitin plasmids were co-transfected in HEK293T cells for 24 h. Cells were then harvested and lysed with NETN buffer. SFB-Mxi1 protein was enriched by S beads co-immunoprecipitation. The ubiquitination-modified lysine (K) sites were identified by mass spectrometry (MS) analysis.

### Viral packaging and infection

This method was carried out as previously described [23–25]. In brief, HEK293T cells were co-transfected with viral packaging plasmids pSPAX2 and pMD2. G as well as control shRNAs or shRNAs against UBE2O, respectively. After 48 h of transfection, the lentivirus supernatants were harvested and filtered. H1299 cells were infected with virus particles in addition with 10 μg/mL Polybrene. Stable cells were screened by puromycin (2 μg/mL) and confirmed by immunoblotting with anti-UBE2O antibody. The shRNAs against UBE2O were as follows:

sh-UBE2O#1: 5-CGATGATTCCTATGGCTTCTA-3;

sh-UBE2O#2: 5-CGGGTCTCTTCTTCGATGATT-3 [26].

### EdU assay

The Cell-Light EdU Apollo567 in vitro kit was purchased from RIBOBIO, and the EdU assay was conducted as per the instruction manual. Briefly, cells transfected with indicated siRNAs or treated with ATO were suspended and seeded in a 96-well plate. After 2 h incubation with 50 μM EdU, cells were washed and fixed with 4 % formaldehyde for 30 min. EdU-Apollo was added for 30 min and cells were treated with 0.5% Triton × 100/PBS for 10 min. Cells were counterstained with DAPI and then detected by fluorescent microscope.

### Flow cytometry analysis

This assay was performed as described previously [25]. Cells transfected with the indicated siRNAs for 24 h were irradiated and collected after another 24 h. The samples were analyzed by flow cytometry.

### CFSE assay

Cells were plated in six-well plates for 24 h and incubated with CFSE (carboxy fluorescein succinimidyl ester) for 10 min. The CFSE labeling was stopped by the treatment with 40% cold bovine serum. Flow cytometry analysis was used to measure cell proliferation.

### Clonogenic cell survival assay

Cells were plated in six-well plates and exposed to irradiation with indicated doses. After being cultured for 14 days, cell clones were fixed with 4% formaldehyde and treated with crystal violet solution for 30 min. Colonies with more than 50 cells were evaluated.

### Neutral Comet assay

The reagent kit (4250-050-K) for neutral comet assay was purchased from Trevigen, and this assay was carried out as per manufacturer’s instructions. Briefly, 4 h after irradiation, cells were collected and then combined with molten LMA agarose and immediately pipetted onto a slide. Subsequently, the slides were immersed and placed in 1× Neutral Electrophoresis Buffer at 21 V for 45 min and then immersed in DNA precipitation solution for 30 min. Samples were stained with SYBR for 10 min and viewed by a fluorescent microscope. The olive tail moment of at least 100 cells was calculated.

### Immunofluorescence staining

Cells were transfected with indicated siRNAs or treated with ATO and then irradiated. After 4 h of irradiation, cells were fixed with paraformaldehyde and then blocked with 5% bovine serum albumin. After being incubated with Rad51 antibody and secondary antibody, samples were washed with PBS buffer for three times. Rad51 foci was detected under a laser confocal microscope. Cells with more than 10 foci were considered positive cells.

### Mouse xenograft models

Experiments in xenograft mouse models were approved by the Medical Ethics Committee of Tongji Medical College, Huazhong University of Science and Technology. This assay was carried out as previously described [23]. Briefly, BALB/c nude mice (5 weeks old) were randomly grouped and injected subcutaneously with 5 × 10^6^ sh-Control, sh-UBE2O#1 or sh-UBE2O#2 H1299 cells. For irradiation treatment, mice were irradiated with 10 Gy when the tumor volume reached 100  mm^3^. For ATO treatment, ATO (2.5 or 5 mg/kg, i.p.) were administered to mice every day when the tumor volume reached 130 mm^3^. Tumor sizes and weights were monitored every 2 or 3 days.

### Immunohistochemical (IHC) staining

The lung adenocarcinoma tissue microarray, which contained 82 paired carcinoma tissues and adjacent tissues, was purchased from Shanghai Outdo Biotech (Shanghai, China). IHC analysis was performed as previously described [23, 27, 28]. Briefly, the tissue section was dewaxed and hydrated, and the endogenous peroxidase was blocked by 3% hydrogen peroxide. The section was blocked with 5% skimmed milk for 1 h and then incubated with the anti-UBE2O antibody (GTX119315) at 4 °C overnight. After that, biotinylated secondary antibody was added for 1 h. For immunohistochemistry scoring, the intensity of staining (0 = negative, 1 = weak, 2 = moderate, 3 = strong) and the percentage of positively stained tumor cells (1 = 0–25%, 2 = 26–50%, 3 = 51–75%, 4 = 75–100%) were used for the quantification. The total IHC score equals the product of the intensity of staining and the percentage of positively stained tumor cells. The total IHC scores ≤6 were defined as low expression, and >6 were defined as high expression.

### Statistical analysis

Each experiment was performed at least three times independently, and the data were shown as mean ± SD unless stated otherwise. The statistical significance between two independent groups was analyzed by unpaired two-tailed Student’s *t* test. Correlations of UBE2O and Mxi1 expression in tumor tissues was analyzed using the Pearson chi-square test. Overall survival analysis was evaluated by the Kaplan–Meier method. *P* value of <0.05 was considered significant.

## Results

### UBE2O is identified as a Mxi1-interacting protein

**Fig. 1:**
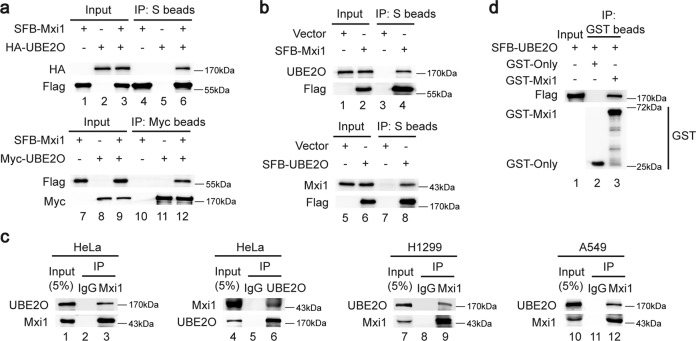
UBE2O interacts with Mxi1 in vitro and in vivo. **a** HEK293T cells transfected with indicated plasmids for 24 h were treated with MG132 (10 μM) for 4 h. Cells were lysed with NETN buffer and then analyzed by co-IP with S beads or Myc agarose followed by western blotting (*n* = 3). **b** HeLa cells were transfected with SFB-Mxi1 or SFB-UBE2O for 24 h and treated with MG132 (10 μM) for 4 h. Cells were collected for co-IP with S beads followed by western blotting (*n* = 3). **c** HeLa, H1299, and A549 cell were treated with MG132 (10 μM) for 4 h and then harvested. Cell lysates were analyzed by co-IP with anti-IgG, Mxi1, or UBE2O antibodies followed by western blotting (*n* = 3). **d** Recombinant GST-only and GST-Mxi1 protein were purified from BL21 bacteria. Cell lysates from HEK293T cells transfected with SFB-UBE2O were incubated with GST or GST-Mxi1 proteins coupled to GST beads overnight and then detected by immunoblotting (*n* = 3).

To better understand how Mxi1 is controlled at the posttranslational level, we previously conducted a tandem affinity purification/MS analysis to identify Mxi1-binding proteins [20]. Of them, CUL1 and β-Trcp2 have been identified as Mxi1-associated proteins previously [20]. In addition, we noticed that UBE2O is a novel candidate Mxi1-interacting partner (Supplementary Fig. 1). To validate this purification result, we employed co-immunoprecipitation experiments and demonstrated that exogenously expressed Mxi1 associated with UBE2O and vice versa (Fig. 1a). Moreover, endogenous UBE2O was co-precipitated in cells ectopically expressing Mxi1 and vice versa (Fig. 1b). Importantly, the interaction between endogenous UBE2O and Mxi1 was also observed in HeLa, H1299, and A549 cells (Fig. 1c). In addition, bacterially expressed GST-tagged Mxi1 bound to UBE2O, again indicating that Mxi1 forms a complex with UBE2O (Fig. 1d). Thus, these results confirmed that UBE2O physically interacts with Mxi1 in cells.

### UBE2O promotes poly-ubiquitination and degradation of Mxi1

**Fig. 2:**
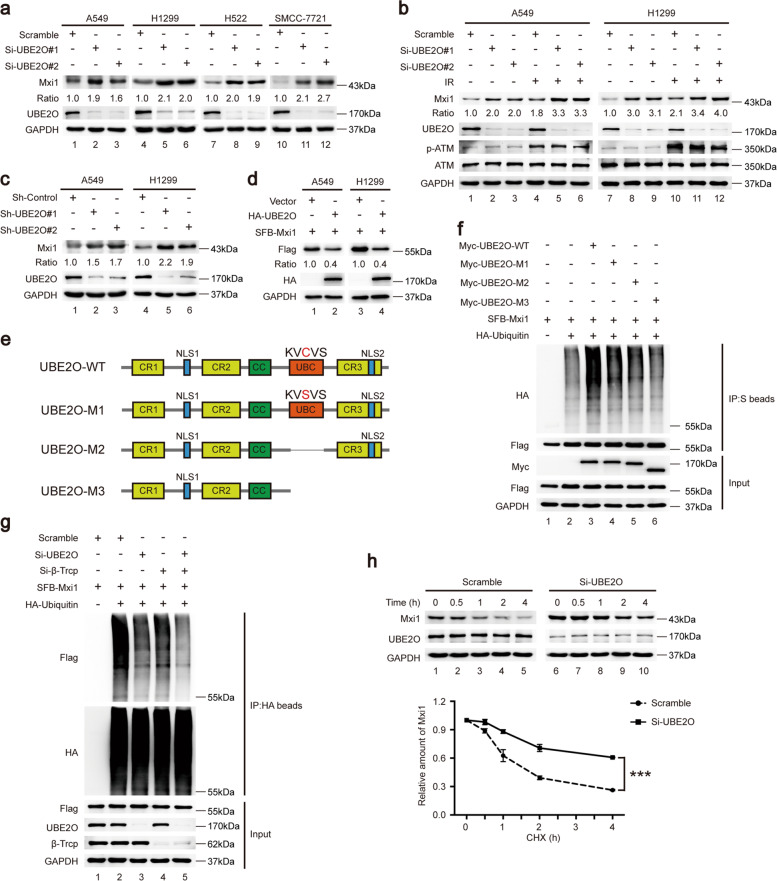
UBE2O negatively controls Mxi1 abundance via the ubiquitin-proteasome pathway. **a** Scramble or UBE2O siRNAs were transfected into lung cancer cells for 48 h. Cells were lysed and then detected by western blotting (*n* = 3). **b** A549 and H1299 cells were transfected with indicated siRNAs for 48 h and then irradiated for 1 h. Cells were lysed and then subjected to western blotting (*n* = 3). p-ATM was used as a positive control upon DNA damage. **c** ShRNAs-mediated knockdown of UBE2O led to increased Mxi1 protein level (*n* = 3). **d** Cells transfected with indicated plasmids for 24 h were collected and then subjected to western blotting (*n* = 3). **e** Schematic presentation of wild-type and mutants UBE2O. **f** HeLa cells were transfected with the indicated plasmids, followed by treatment with MG132 (10 μM) for 4 h prior to collection. The lysates were incubated with S protein beads and then subjected to immunoblotting (*n* = 3). **g** HeLa cells were transfected with indicated siRNAs for 24 h, and then co-transfected with SFB-Mxi1 and HA-Ubiquitin plasmids for another 24 h. After treatment with MG132 (10 μM) for 4 h, cells were collected for co-IP with HA beads, and then subjected to immunoblot analysis (*n* = 3). **h** Upper panel: Scramble or UBE2O siRNAs were transfected into HeLa cells for 48 h. Cells were then treated with cycloheximide (CHX, 100 μg/mL) and collected for immunoblot analysis at the indicated time points. Lower panel: quantification of Mxi1 band intensity was presented (*n* = 3). ***P < 0.001.

Given that UBE2O is an E2/E3 hybrid ubiquitin-protein ligase and has been reported to mediate ubiquitination of several proteins, we therefore speculated that Mxi1 might be a ubiquitination substrate of UBE2O. As shown in Fig. 2a, b, depletion of UBE2O with specific siRNAs led to an elevation in endogenous Mxi1 protein levels in different cancer cells under steady-state conditions and after DNA damage. ShRNAs-mediated UBE2O knockdown also upregulated Mxi1 protein expression (Fig. 2c). In keeping with this idea, the ectopic expression of UBE2O decreased the protein level of Mxi1 in A549 and H1299 cells (Fig. 2d), indicating that UBE2O is involved in controlling Mxi1 stability. Next, to explore whether UBE2O governs the stability of Mxi1 through ubiquitination, we generated three UBE2O mutants with inactivated or deleted UBC domain and found that wild-type (WT) UBE2O could dramatically promotes the poly-ubiquitination of Mxi1 in comparison with the catalytically inactive UBE2O mutant (M1, C1040S) and the UBC domain-deleted UBE2O mutants (M2 and M3) (Fig. 2e, f), indicating that UBE2O may be the major E2/E3 ubiquitin ligase for Mxi1. Consistently, knockdown of UBE2O greatly reduced Mxi1 poly-ubiquitination in vivo (Fig. 2g). Notably, β-Trcp knockdown also decreased the poly-ubiquitination of Mxi1, which is consistent with our previous report [20]. More importantly, compared with knockdown of UBE2O or β-Trcp individually, depletion of UBE2O and β-Trcp simultaneously exhibited a more pronounced effect on Mxi1 poly-ubiquitination (Fig. 2g), suggesting that UBE2O and β-Trcp synergistically target Mxi1 for poly-ubiquitination. In agreement with this idea, loss of UBE2O resulted in an extended half-life of endogenous Mxi1 (Fig. 2h). These results demonstrate that UBE2O is a bona fide E2/E3 ligase that targets Mxi1 for ubiquitination and degradation in lung cancer cells.

### K46 of Mxi1 is the major ubiquitination target residue of UBE2O

**Fig. 3:**
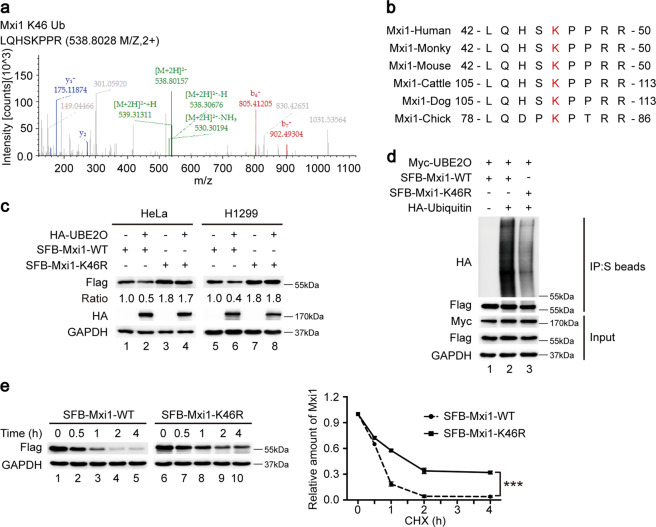
K46 of Mxi1 is the major ubiquitination target residue of UBE2O. **a** Mass spectrometry analysis was used for identifying the ubiquitination sites in Mxi1. The di-glycine-modified K46 in Mxi1 was detected, and the corresponding peptide was LQHSKPPR. **b** Alignment of the K46 corresponding amino acids sequences in Mxi1 from different species. **c** HeLa (upper panel) or H1299 (lower panel) cells were co-transfected with vector expressing HA-UBE2O together with indicated plasmids. After 24 h, cells were lysed for immunoblot analysis (*n* = 3). **d** HeLa cells were co-transfected with the indicated plasmids for 24 h, followed by treatment with MG132 (10 μM) for 4 h prior to collection. Cells lysates were subjected to co-IP with S beads followed by western blotting (*n* = 3). **e** Left panel: HeLa cells were transfected with SFB-Mxi1-WT or SFB-Mxi1-K46R plasmid for 24 h, and then treated with cycloheximide (CHX, 20 μg/mL) for the indicated times before harvesting. Cells lysates were analyzed by immunoblotting. Right panel: Mxi1 band intensity was measured by ImageJ software (*n* = 3). ***P < 0.001.

To identify the potential ubiquitination site of Mxi1 by UBE2O, we performed a MS analysis and found the lysine 46 (K46) site in Mxi1 had a di-glycine modification (Fig. 3a), indicating that K46 may be the ubiquitination site. Moreover, we noted that the K46 residue of Mxi1 is highly conserved among various species (Fig. 3b). To determine whether this putative site is responsible for Mxi1 degradation, we mutated the K46 residue to arginine (termed the K46R mutant). As shown in Fig. 3c, the ectopic expression of UBE2O led to a decrease of WT but not the K46R mutant of Mxi1. In addition, the K46R mutant dramatically reduced poly-ubiquitination of Mxi1 in the presence of UBE2O (Fig. 3d). These findings suggest that K46 site is responsible for the ubiquitination and stability of Mxi1 by UBE2O. Consistent with these findings, the half-life of the Mxi1 K46R mutant was longer than that of WT Mxi1 (Fig. 3e). Accordingly, our data suggest that K46 of Mxi1 is the major ubiquitination target residue of UBE2O.

### Loss of UBE2O impairs tumorigenesis and radioresistance in lung cancer in vitro and in vivo

**Fig. 4:**
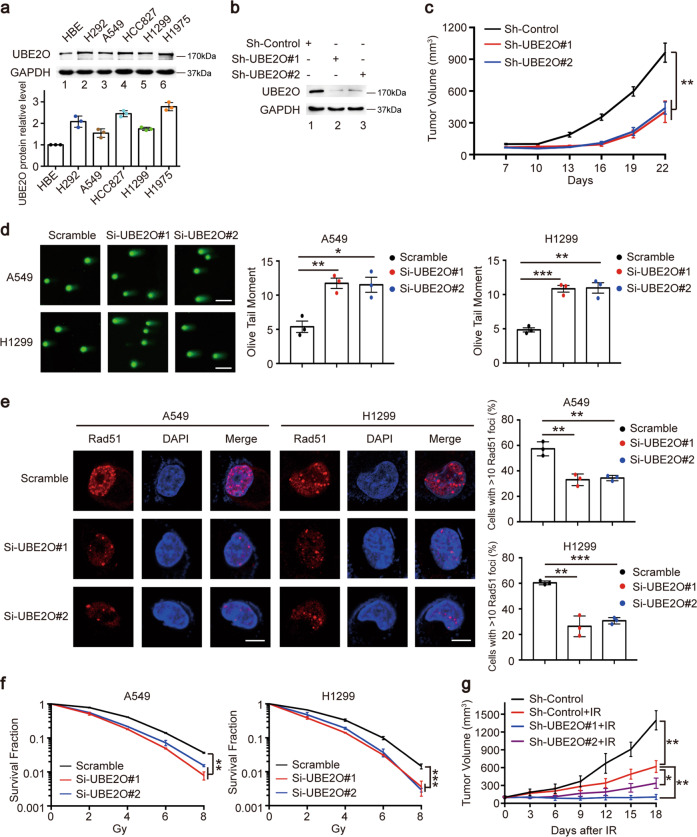
Loss of UBE2O reduces tumor growth and enhances radiosensitivity in lung cancer in vitro and in vivo. **a** Upper panel: cell lysates were analyzed by immunoblotting with anti-UBE2O antibody. Lower panel: quantification of UBE2O band intensity was shown (*n* = 3). **b** H1299 cells stably expressing sh-Control or sh-UBE2O were collected and examined the knockdown efficiency of UBE2O by western blotting (*n* = 3). **c** Nude mice were subcutaneously injected with H1299 cells stably expressing sh-Control or sh-UBE2O. The tumor volumes were measured every 3 days (*n* = 4 mice/group). Data were shown as mean ± SEM. **P < 0.01. **d** Left panel: representative images of the comet tail. Scale bar, 50 μm. Right panel: quantification of the olive tail moment in each group were shown (*n* = 3). *P < 0.05, **P < 0.01, ***P < 0.001. **e** Left panel: representative immunostaining images of Rad51 foci. A cell containing 10 or more foci was considered as a foci-positive cell. Scale bar, 10 μm. Right panel: the percentage of Rad51 foci-positive cells in each group were shown (*n* = 3). **P < 0.01, ***P < 0.001. **f** H1299 and A549 cells were transfected with indicated siRNAs and irradiated at different doses as indicated respectively. After 14 days, cell colonies were fixed and counted, and the survival fraction was calculated (*n* = 3). **P < 0.01, ***P < 0.001. **g** The growth curves of the xenograft tumors for each group are represented. H1299 cells stably expressing sh-Control or sh-UBE2O were subcutaneously injected in the right hind limb of nude mice, respectively. The mice were irradiated with 10 Gy when the tumor volume reached 100 mm^3^. Data were shown as mean ± SEM (*n* = 5 mice/group). *P < 0.05, **P < 0.01.

Our previous study has clearly shown that Mxi1 is downregulated and inhibits lung cancer cell proliferation [20]. As mentioned above, UBE2O negatively regulates the abundance of Mxi1. Therefore, we surmised that UBE2O might act as an oncoprotein in lung cancer. As shown in Fig. 4a, UBE2O protein levels were upregulated in five lung cancer cell lines compared with that in normal human epithelial cell line HBE. To determine the roles of UBE2O in lung cancer progression, we performed cell growth and proliferation assays and showed that UBE2O silencing with two different specific siRNAs led to a greater inhibition of cell growth and colony formation ability in A549 and H1299 cells (Supplementary Fig. 2A, B). In addition, knockdown of UBE2O significantly decreased the percentage of EdU-positive cells (Supplementary Fig. 2C). These results demonstrate that UBE2O indeed promote cell proliferation in vitro. To explore whether UBE2O facilitates tumorigenesis in vivo, we generated H1299 cells stably transfected with Sh-Control or Sh-UBE2O (Fig. 4b), and then performed a subcutaneous xenograft experiment in nude mice. As shown in Fig. 4c, mice inoculated with Sh-UBE2O cells exhibited slower tumor growth compared with those inoculated with Sh-Control cells. These results strongly support the idea that UBE2O overexpression leads to lung cancer progression in vitro and in vivo.

We have previously found that overexpression of Mxi1 enhanced the radiosensitivity in lung cancer cells [20]. Since UBE2O controls the accumulation of Mxi1, we next investigated whether UBE2O is associated with radiosensitivity of lung cancer. To this point, we first performed the neutral comet assay and demonstrated that knockdown of UBE2O significantly increased the length and DNA content in the comet tails (Fig. 4d). It has been well-established that Rad51 foci formation is considered the readout of homologous recombination repair in response to DNA damage [29]. Consistent with the neutral comet assay, DNA damage-induced Rad51 foci formation was drastically decreased when UBE2O was knocked down (Fig. 4e), indicating that loss of UEB2O can accelerate DNA damage and impair DNA repair in lung cancer. Furthermore, clonogenic survival assays revealed that UBE2O silencing enhanced the lung cancer cells radiosensitivity (Fig. 4f). In addition, we did not observe a significant change in irradiation-induced G2/M arrest or early apoptosis in UBE2O-depleted lung cancer cells (Supplementary Fig. 2D, E). These data strongly indicate that UBE2O indeed results in radioresistance in vitro. To further confirm these findings in vivo, we conducted subcutaneous xenograft experiment in nude mice. As shown in Fig. 4g, the tumor growth in the irradiated Sh-Control group was significantly slower than that in unirradiated Sh-Control group, suggesting that irradiation alone is effective. More importantly, we observed that the tumor growth in sh-UBE2O groups combined with irradiation was significantly decreased compared with that in Sh-Control group with irradiation. Collectively, these results suggest that UBE2O depletion impairs tumorigenesis and enhances the radiosensitivity in lung cancer in vitro and in vivo.

### Pharmacological blockade of UBE2O with ATO suppresses tumorigenesis and enhanced the radiosensitivity in lung cancer in vitro and in vivo

**Fig. 5:**
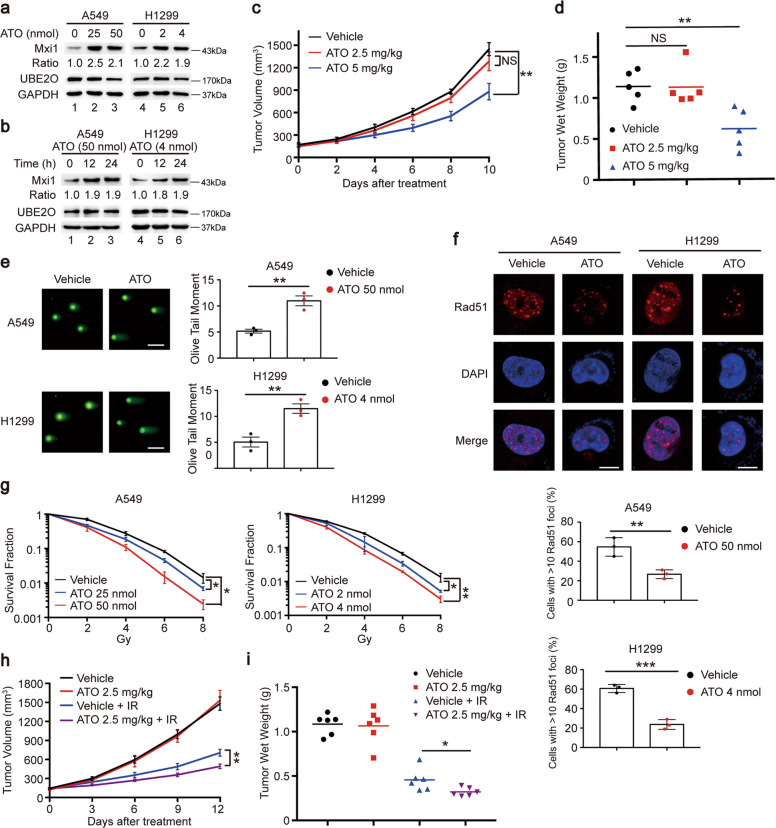
Arsenic trioxide (ATO) impairs tumor growth and radioresistance in lung cancer in vitro and in vivo. **a** A549 and H1299 cells were treated with ATO at the indicated concentration for 24 h and then collected for immunoblot analysis (*n* = 3). **b** A549 and H1299 cells were treated with ATO for 12 h or 24 h with the indicated concentration. Western blotting was used for analyzing the protein level of Mxi1 (*n* = 3). **c** The volume of the xenograft tumors in each group are shown. Nude mice subcutaneously injected with H1299 cells were treated with ATO (Intraperitoneal injection, daily, 2.5 mg/kg or 5 mg/kg) when the tumor volumes reached 130 mm^3^ (*n* = 5 mice/group). Data were presented as mean ± SEM. **P < 0.01. **d** The tumor weights of each group were presented (*n* = 5 mice/group). **P < 0.01. **e** Left panel: representative images of comet tail. Scale bar, 50 μm. Right panel: quantification of the olive tail moment was shown (*n* = 3). **P < 0.01. **f** Upper panel: representative images of Rad51 foci. Scale bar, 10 μm. Lower panel: the percentage of Rad51 foci-positive cells in each group were shown (*n* = 3). **P < 0.01, ***P < 0.001. **g** A549 and H1299 cells treated with ATO at the indicated concentration for 24 h were irradiated at the indicated doses. The survival fraction was calculated after 2 weeks (*n* = 3). *P < 0.05, **P < 0.01. **h** Tumor growth curves of each group were shown. Nude mice were subcutaneously injected with H1299 cells and treated with irradiation only, ATO only or ATO plus 10 Gy irradiation (*n* = 6 mice/group). Data were shown as mean ± SEM. **P < 0.01. **i** The tumor weights in each group are shown (*n* = 6 mice/group). *P < 0.05.

The biological functions showed that UBE2O plays oncogenic roles in lung cancer and is expected to be an attractive target for anticancer and radiosensitization therapeutics. It has been reported that ATO can inhibit UBE2O activity by crosslinking adjacent cysteines in its catalytic domain [13]. Therefore, we investigated whether blockade of UBE2O with ATO displays effects similar to that of UEB2O deficiency. We first measured the IC50 of ATO at 24 h in A549 and H1299 cells (Supplementary Fig. 3A). As expected, ATO treatment increased the protein levels of Mxi1 in a concentration- and time-dependent manner (Fig. 5a, b). Furthermore, ATO was able to impede lung cancer cell growth and colony formation (Supplementary Fig. 3B, C). In line with this idea, EdU and CFSE assays showed that ATO treatment significantly reduced cell proliferation in lung cancer (Supplementary Fig. 3D, E). These results clearly demonstrated that ATO could indeed suppress tumorigenesis in lung cancer in vitro. To further investigate the antitumor effect of ATO in vivo, we subcutaneously implanted H1299 cells into nude mice and treated them daily with saline or ATO by intraperitoneal injection. As shown in Fig. 5c, d, 2.5 mg/kg concentration of ATO had no effect on tumor growth and weight, however, 5 mg/kg concentration of ATO remarkably reduced tumor growth and weight, suggesting that ATO treatment decreases tumor growth in a concentration-dependent manner. Therefore, these findings provide evidence that the blockade of UBE2O with ATO restrains tumorigenesis in lung cancer in vitro and in vivo.

Given that UBE2O depletion enhanced the radiosensitivity of lung cancer, we therefore wondered whether pharmacological inhibition of UBE2O with ATO had a similar effect. To verify our hypothesis, we performed the neutral comet and Rad51 foci experiments and found that ATO treatment significantly increased the length and DNA content in the comet tails as well as impaired the formation of Rad51 foci (Fig. 5e, f). In agreement with these data, clonogenic survival assay showed that ATO blockade remarkably enhanced cell radiosensitivity to irradiation (Fig. 5g). More importantly, we observed that although single ATO treatment with a concentration of 2.5 mg/kg could not inhibit tumor growth, however, the combination of irradiation and the same drug concentration of ATO reduced both the tumor growth and weight more notably compared with the group treated with irradiation only (Fig. 5h, i), suggesting that ATO treatment effectively enhanced lung cancer radiosensitivity in vivo. Collectively, these results demonstrate that ATO treatment can overcome radioresistance in lung cancer in vitro and in vivo, indicating that ATO may be a potential radiosensitizing drug in lung cancer radiotherapy.

### UBE2O exerts the biological effects through negative regulation of Mxi1 in lung cancer cells

**Fig. 6:**
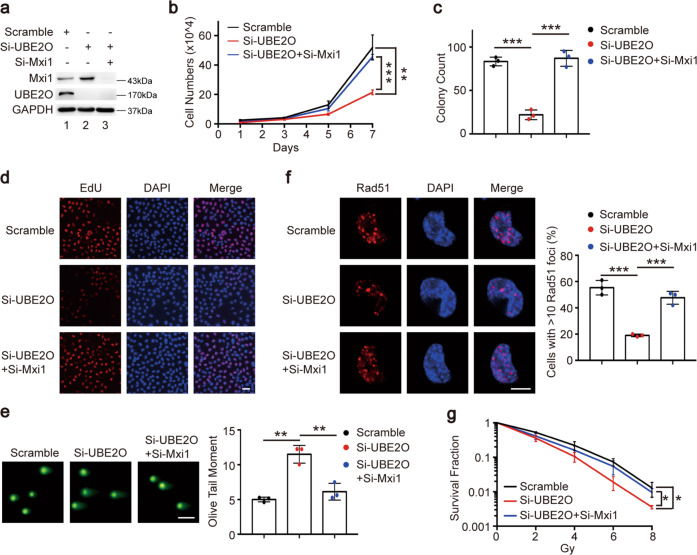
UBE2O promotes lung cancer cell proliferation and radioresistance through negative regulation of Mxi1. **a** H1299 cells transfected with indicated siRNAs were lysed and analyzed by immunoblotting (*n* = 3). **b** H1299 cells transfected with indicated siRNAs were seeded in six-well plates with 1 × 10^4^ cells and counted every other day (*n* = 3). **P < 0.01, ***P < 0.001. **c** H1299 cells transfe**c**ted with the indicated siRNAs were seeded in six-well plates with 200 cells/well. Two weeks later, cell colonies were fixed and counted (up to 50 cells) (*n* = 3). ***P < 0.001. **d** Representative images of EdU labeled cells in each group. H1299 cells were transfected with the indicated siRNAs for 48 h, and then incubated with EdU for 2 h (*n* = 3). Scale bar, 50 μm. **e** Left panel: representative comet tail images in each group. Scale bar, 50 μm. Right panel: quantification of the olive tail moment was shown (*n* = 3). **P < 0.01. **f** Left panel: representative images of Rad51 foci in each group. Scale bar, 10 μm. Right panel: the percentage of Rad51 foci-positive cells in each group were shown (*n* = 3). ***P < 0.001. **g** H1299 cells were transfected with the indicated siRNAs and then irradiated at various doses. Two weeks later, the colonies were calculated (up to 50 cells). The survival fraction was shown as indicated (*n* = 3). *P < 0.05.

Since Mxi1 is a ubiquitination substrate of UBE2O, we determined whether Mxi1 is required for the functions of UBE2O in lung cancer. H1299 cells were transfected with siRNAs targeting UBE2O, Mxi1 or both (Fig. 6a). As seen in Fig. 6b, c, Mxi1 silencing reversed the defects in cell growth and colony formation in UBE2O deficiency cells. In line with this notion, the EdU assay clearly showed Mxi1 knockdown rescued the cell proliferation defect in UBE2O deficiency cells (Fig. 6d). Moreover, the increased comet tail and the reduced Rad51 foci formation caused by UBE2O silencing were restored when Mxi1 was co-depleted (Fig. 6e, f). Consistently, Mxi1 knockdown also rescued the defect in cell survival after irradiation in UBE2O-depleted cells (Fig. 6g). Collectively, our results indicate that UBE2O promotes cell proliferation and radioresistance via its ability to degrade Mxi1 in lung cancer cells.

### UBE2O overproduced correlates with Mxi1 downregulation and predicts poor prognosis in lung cancer

**Fig. 7:**
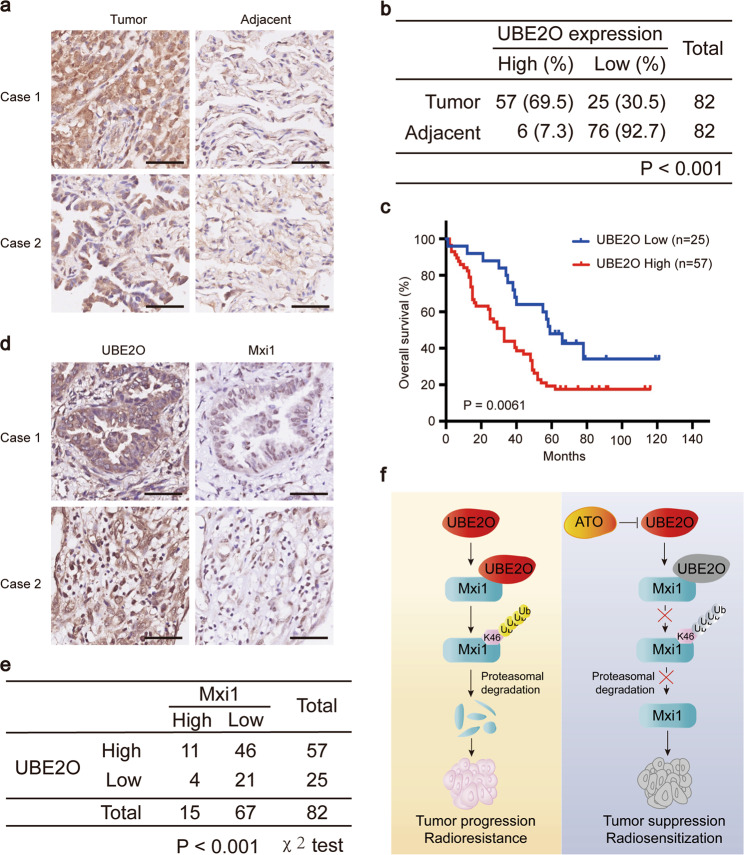
UBE2O overexpression predicts poor prognosis and correlates with Mxi1 downregulation in lung cancer patients. **a** Representative IHC staining image for UBE2O in lung adenocarcinoma and paired adjacent lung tissue. Scale bar, 50 μm. **b** Quantification of UBE2O protein levels in a lung cancer tissue microarray. **c** Overall survival analysis between UBE2O high and low-expression lung cancer patients by Kaplan–Meier method. **d** Representative IHC staining images for UBE2O and Mxi1 in two lung adenocarcinoma cases. Scale bar, 50 μm. **e** The association between UBE2O and Mxi1 protein level in 82 lung adenocarcinoma tissues. **f** A proposed model how UBE2O regulates Mxi1 in lung cancer. Left panel: UBE2O targets Mxi1 for ubiquitination and degradation at K46 site, therefore leading to tumor progression and radioresistance. Right panel: pharmacological inhibition of UBE2O with ATO blocks the degradation of Mxi1, ultimately resulting in tumor suppression and radiosensitization.

Our previous study has shown that Mxi1 is downregulated and associated with adverse clinical outcomes in lung cancer patients [20]. Since UBE2O negatively regulates the protein levels of Mxi1, we speculated that UBE2O might be overexpressed in lung cancer. To confirm whether UBE2O is indeed upregulated in lung cancer, we validated the specificity of anti-UBE2O antibody and then detected the expression of UBE2O in 82 pairs of lung adenocarcinoma tissues and adjacent tissues using IHC staining. We found that UBE2O is highly expressed in lung adenocarcinoma tissues than that in adjacent tissues (Fig. 7a, b and Supplementary Fig. 4A, B). To determine whether the expression of UBE2O is related to the clinical outcome of lung cancer patients, we divided the 82 lung cancer patients into UBE2O high and low-expression group based on the intensity of staining and the percentage of positively stained tumor cells. As shown in Fig. 7c, the high expression of UBE2O was associated with shorter overall survival in lung cancer patients (*P* < 0.05). Importantly, we analyzed the association between UBE2O and Mxi1 expression in the paired lung adenocarcinoma tissues and found that there was a negative correlation between UBE2O and Mxi1 protein levels (*P* < 0.001) (Fig. 7d, e). Taken together, these data demonstrate that UBE2O overproduction may lead to Mxi1 downregulation and predict adverse prognosis in lung cancer patients.

## Discussion

The downregulation of Mxi1 expression has been found in human lung cancer [20], but the mechanisms of Mxi1 downregulation has not been fully understood. In this study, we demonstrate that UBE2O targets Mxi1 for ubiquitination and degradation, which is a new mechanism for Mxi1 regulation in lung cancer. Moreover, genetical and pharmacological inhibition of UBE2O impairs tumorigenesis and radioresistance in a Mxi1-dependent manner in lung cancer in vitro and in vivo. Furthermore, UBE2O overexpression predicts poor clinical outcomes and correlates with Mxi1 downregulation in lung cancer tissues, indicating that targeting the UBE2O/Mxi1 axis may be an attractive therapeutic strategy for treating lung cancer.

A growing body of evidence has shown that Mxi1 acts as an antagonist of proto-oncogene c-Myc through competing for Max and repressing the transcriptional activity of c-Myc [17]. We previously reported that Mxi1 is a labile protein which can be ubiquitinated and degraded by β-Trcp [20]. However, we still observed the poly-ubiquitination of Mxi1 when the β-Trcp-binding motif in Mxi1 was mutated, indicating the existence of additional E3 ligase controlling Mxi1 destruction. In this study, using a proteomic interaction screen, we identified E2/E3 hybrid ubiquitin-protein ligase UBE2O as a new interacting protein of Mxi1. We further showed that UBE2O binds to and ubiquitinates Mxi1 at lysine 46 residue, therefore leading to the degradation of Mxi1 via UPS, indicating UBE2O is a critical negative regulator of Mxi1 at the posttranscriptional level. In addition, we also demonstrated that UBE2O acts synergistically with β-Trcp to ubiquitinate Mxi1. Thus, our work provides evidence that UBE2O is a novel ubiquitin ligase which is responsible for the degradation of Mxi1. It has been documented that UBE2O is frequently amplified or overexpressed in several types of human cancer, which include bladder cancer, breast cancer, and liver cancer [13, 30]. Our clinical data clearly show that UBE2O protein is overproduced and inversely correlated with Mxi1 expression in lung cancer. Considering that UBE2O negatively controls the abundance of Mxi1 at translational level, our results strongly suggest that UBE2O overexpression may result in the downregulation of Mxi1 in lung cancer.

UBE2O has been reported to play essential roles in cell proliferation and apoptosis, cellular clock function, and metabolic homeostasis [26, 31–33]. For instance, UBE2O has been found to control the stability of MLL/COMPASS and play an oncogenic role in MLL-rearranged leukemia [26]. Xu et al. showed that UBE2O promotes the poly-ubiquitination of c-Maf to inhibit the growth of multiple myeloma cells [31]. These studies indicate that UBE2O may play different roles in tumor progression, depending on the cancer type or targeted substrate. Our data clearly show that UBE2O silencing inhibits lung cancer cell growth and proliferation in vitro and in vivo, suggesting that UBE2O may function as an oncoprotein that promotes lung cancer progression. Importantly, we first revealed a novel function for UBE2O in promoting radioresistance. UBE2O depletion overcomes lung cancer radioresistance in vitro and in vivo, indicating that UBE2O may be an attractive radiosensitization target in lung cancer. Notably, inactivation of UBE2O with ATO dramatically upregulated the protein levels of Mxi1 and enhanced radiosensitivity in lung cancer, suggesting that ATO exerts radiosensitization effect via the inhibition of the UBE2O-Mxi1 axis. Furthermore, our rescue experiments showed that the inhibiting effects of UBE2O depletion on the proliferation and radioresistance of lung cancer cells can be restored by Mxi1 knockdown. These results strongly imply that Mxi1 is a major downstream effector of UBE2O in lung cancer. Given that the anticancer and radiosensitization potential of UBE2O blockade, our data provide a strong rationale that the development of UBE2O specific inhibitor may be a potential therapeutic strategy for treating lung cancer.

In summary, our work identifies UBE2O as a novel E3 ubiquitin ligase targeting Mxi1 for ubiquitination and destruction, leading to tumorigenesis and radioresistance in vitro and in vivo, as proposed in Fig. 7f. We also found that genetical and pharmacological inhibition of UBE2O impair tumorigenesis and radioresistance in a Mxi1-dependent manner in lung cancer. These data not only shed light on the molecular mechanism underlying how Mxi1 is regulated by UBE2O but also imply that UBE2O is a potential drug target for lung cancer intervention.

## Supplementary information

Supplemental Material

Supplementary Figure 1

Supplementary Figure 2

Supplementary Figure 3

Supplementary Figure 4
