# Supplementary material for: UBE2O targets Mxi1 for ubiquitination and degradation to promote lung cancer progression and radioresistance
Source: Cell Death Differ. 2020 Sep 8;28(2):671–84. doi: 10.1038/s41418-020-00616-8 (PMC7862231; doi:10.1038/s41418-020-00616-8)
Supplement: Supplementary file 1 — Supplemental Material [file 41418_2020_616_MOESM1_ESM.docx]

**Supplementary Fig. 1 The Mxi1-interacting proteins and peptide numbers are shown.** Mxi1-interacting proteins were identified by TAP/MS analysis. The name and peptide numbers of identified proteins were listed.

**Supplementary Fig. 2 UBE2O silencing inhibits lung cancer cell growth and proliferation.** **a** Cells transfected with indicated siRNAs were seeded in 6-well plates with 1×10^4^ cells/well and calculated every other day (n = 3). *** P < 0.001. **b** Cells were transfected with indicated siRNAs and then seeded in 6-well plates with 200 cells/well. Two weeks later, the colonies (up to 50 cells) were counted (n = 3). ** P < 0.01, *** P < 0.001. **c** Representative images of EdU labeled cells were shown (n = 3). Scale bar: 50 μm. **d** H1299 cells transfected with indicated siRNAs for 24 h was irradiated and then incubated for another 24 h. Cells were collected for cell cycle analysis (n = 3). * P < 0.05. **e** H1299 cells were transfected with indicated siRNAs for 24 h and then irradiated. Another 24 h later, cells were harvested and stained with annexin V-EGFP/propidium iodide and then analyzed with flow cytometry (n = 3). * P < 0.05.

**Supplementary Fig. 3 Arsenic trioxide (ATO) impedes cell growth and proliferation in lung cancer. a** Measurement of the IC50 of ATO in A549 and H1299 cells by CCK8 assay. **b** A549 and H1299 cells were treated with ATO at the indicated concentration for 24 h and counted every other day (n = 3). *** P < 0.001. **c** A549 and H1299 cells were treated with ATO at the indicated concentration for 24 h. Two weeks later, the colonies (up to 50 cells) were calculated (n = 3). ** P < 0.01, *** P < 0.001. **d** Representative images of EdU labeled A549 and H1299 cells treated with ATO at the indicated concentration for 24 h (n = 3). Scale bars: 50 μm. **e** A549 and H1299 cells treated with ATO at the indicated concentration for 24 h were incubated with CFSE for 10 min and then collected for flow cytometry analysis (n = 3).

**Supplementary Fig. 4 Validation of the specificity of UBE2O antibody for immunohistochemical (IHC) analysis. a** Immunochemistry using the anti-UBE2O antibody of scramble or UBE2O knockdown lung cancer cells from cultures on glass slides. Scale bar, 50 μm. **b** Representative IHC staining image for UBE2O in xenograft tumors expressing sh-Control or sh-UBE2O. Scale bar, 50 μm.
