# Supplementary figures and images for: UBE2O targets Mxi1 for ubiquitination and degradation to promote lung cancer progression and radioresistance
Source: Cell Death Differ. 2020 Sep 8;28(2):671–84. doi: 10.1038/s41418-020-00616-8 (PMC7862231; doi:10.1038/s41418-020-00616-8)

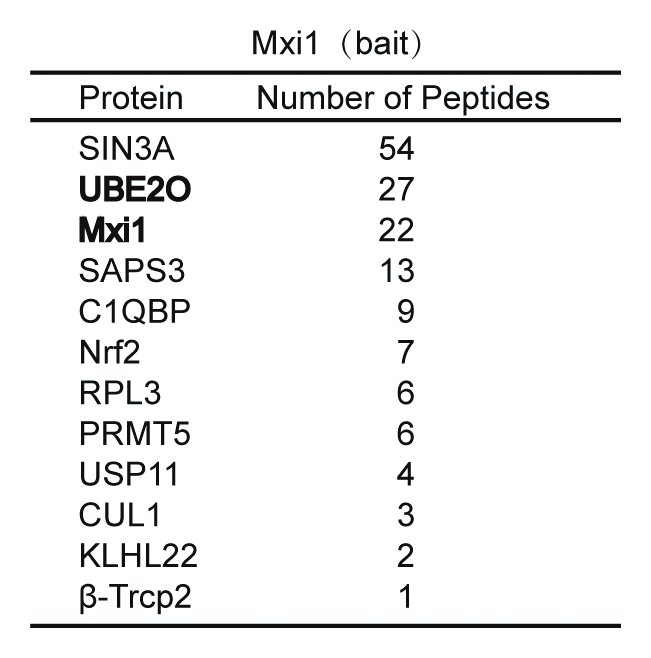

Supplement: Supplementary file 2 — Supplementary Figure 1 [file 41418_2020_616_MOESM2_ESM.tif]

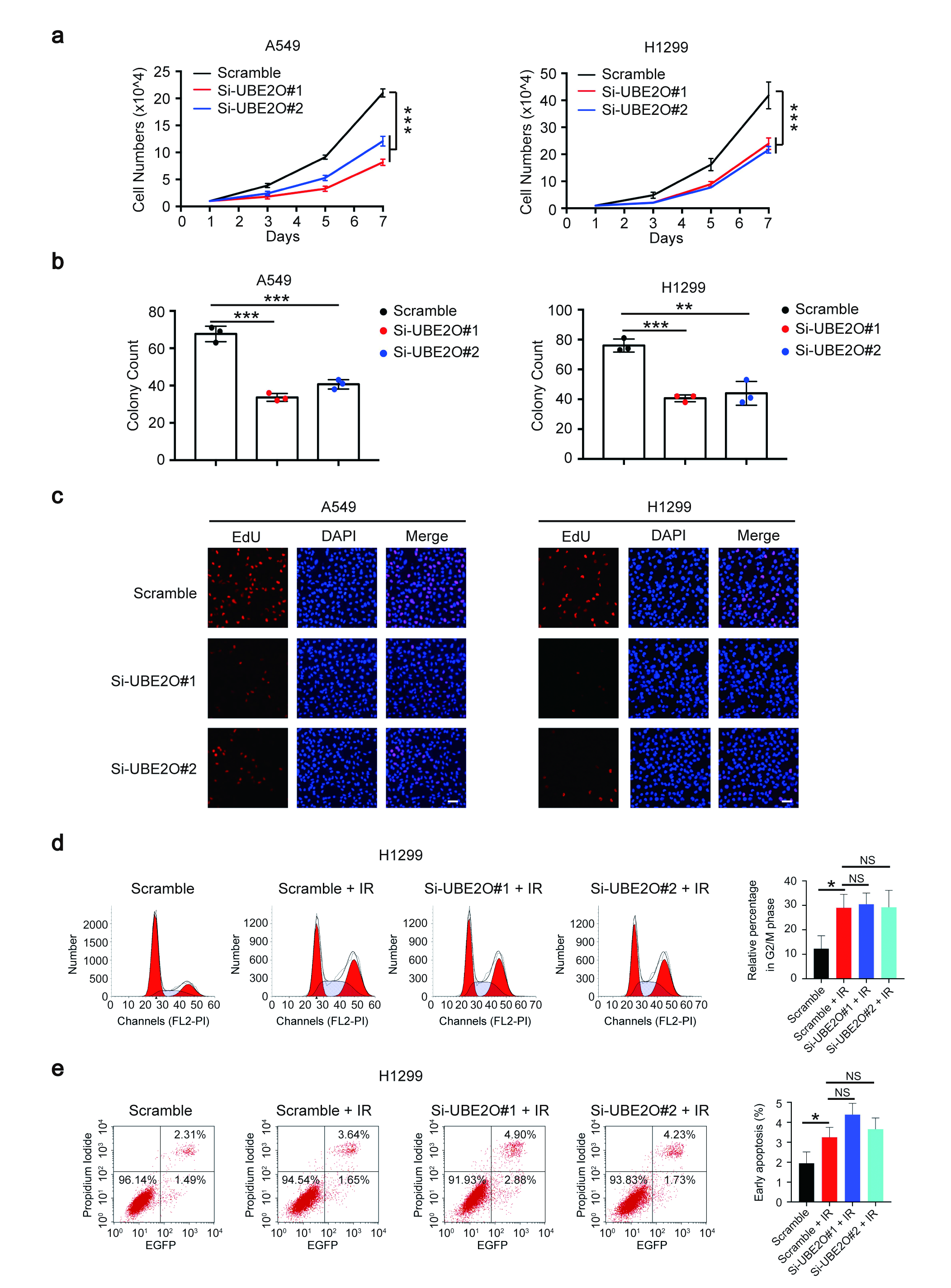

Supplement: Supplementary file 3 — Supplementary Figure 2 [file 41418_2020_616_MOESM3_ESM.tif]

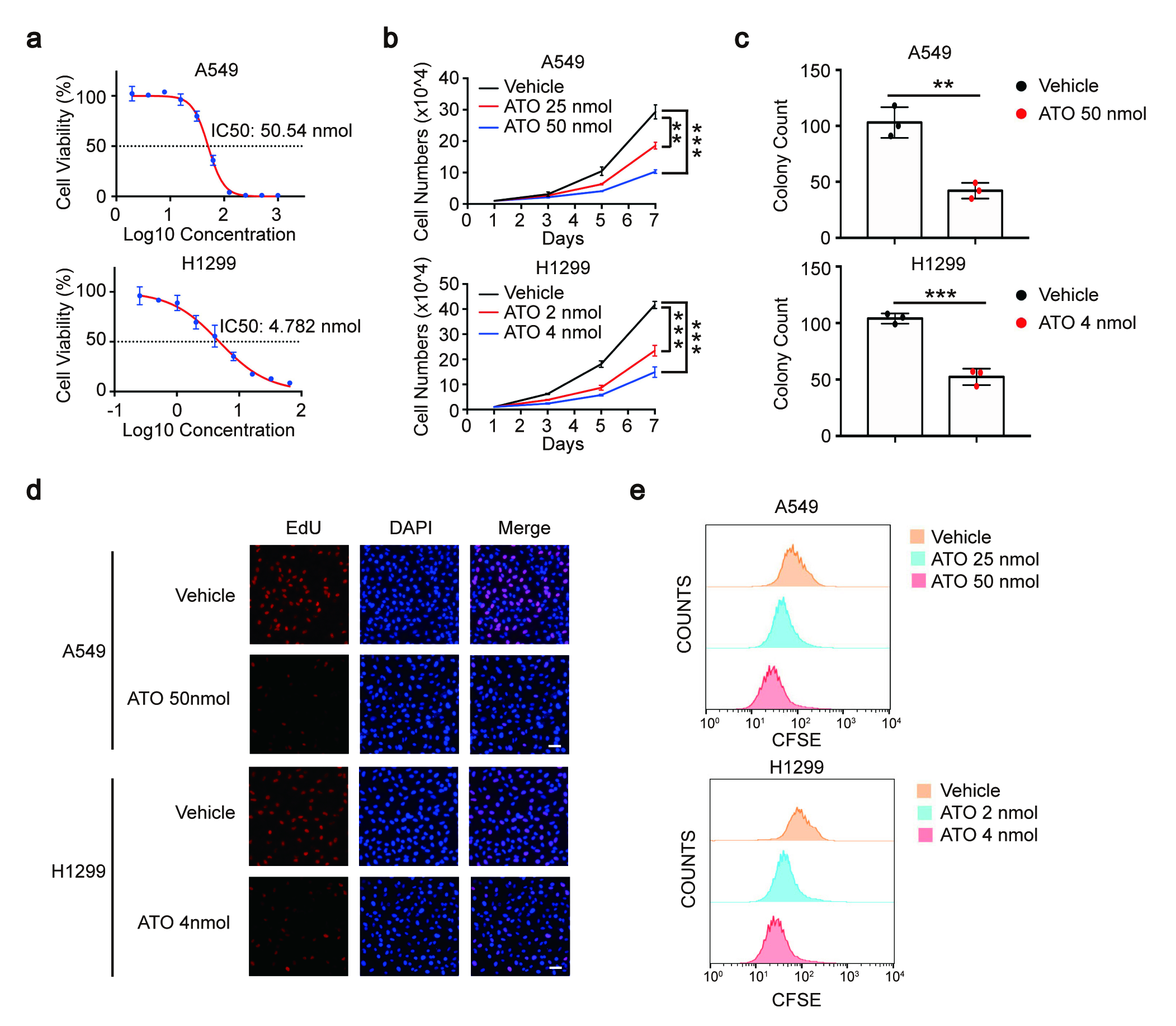

Supplement: Supplementary file 4 — Supplementary Figure 3 [file 41418_2020_616_MOESM4_ESM.tif]

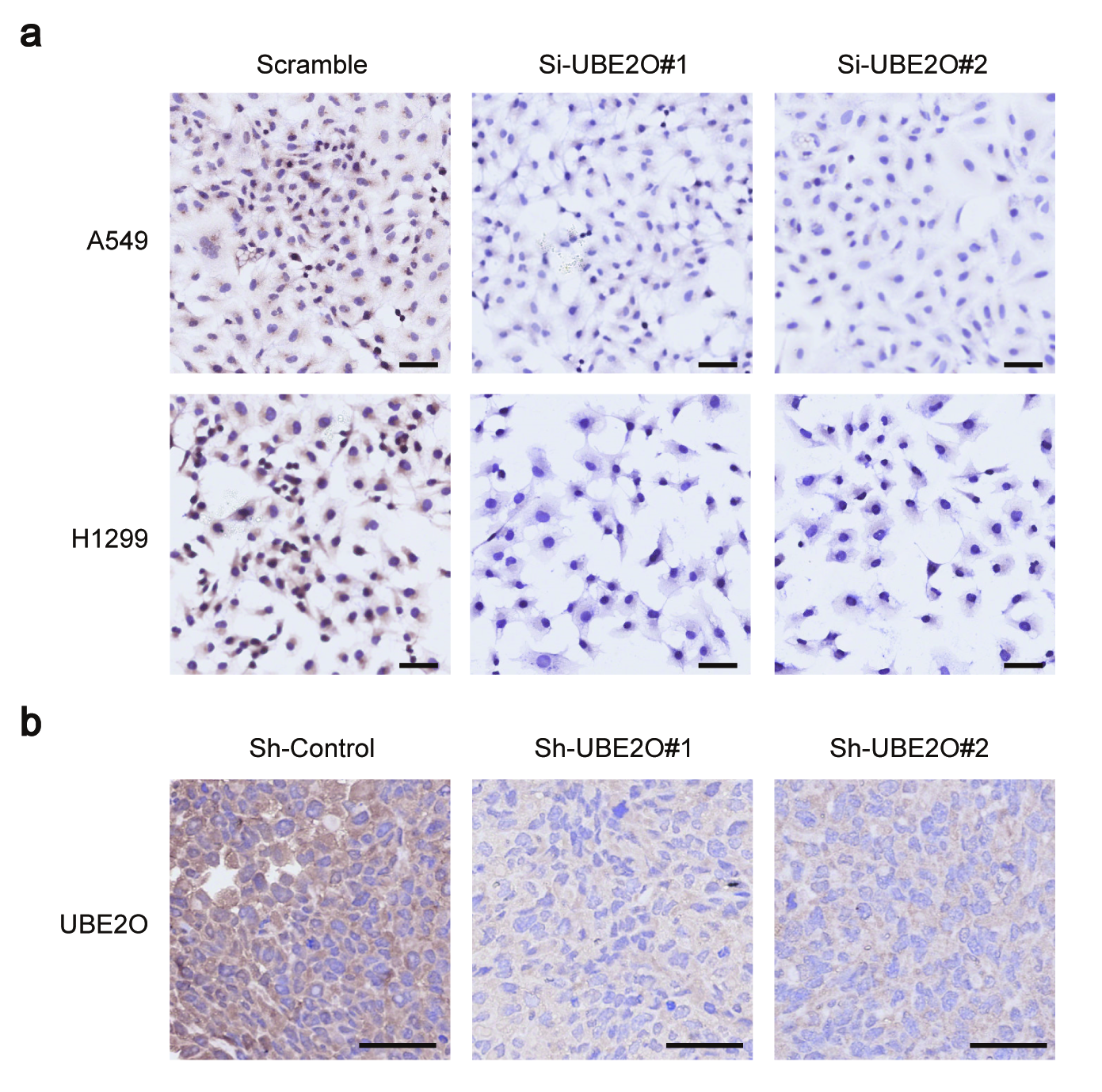

Supplement: Supplementary file 5 — Supplementary Figure 4 [file 41418_2020_616_MOESM5_ESM.tif]
